# The Pragmatic Return to Effective Dental Infection Control Through Triage and Testing (PREDICT) Study: Protocol for a Prospective Clinical Study in the National Dental Practice–Based Research Network

**DOI:** 10.2196/38386

**Published:** 2022-08-31

**Authors:** Janine Fredericks-Younger, Daniel H Fine, Gayathri Subramanian, Modupe O Coker, Cyril Meyerowitz, Patricia Ragusa, Veerasathpurush Allareddy, Mary Ann McBurnie, Ellen Funkhouser, Maria Laura Gennaro, Cecile A Feldman

**Affiliations:** 1 School of Dental Medicine Rutgers University Newark, NJ United States; 2 Eastman Institute for Oral Health University of Rochester Rochester, NY United States; 3 Department of Orthodontics College of Dentistry University of Illinois at Chicago Chicago, IL United States; 4 Center for Health Research Kaiser Permanente Northwest Portland, OR United States; 5 Department of Medicine University of Alabama Birmingham, AL United States; 6 Public Health Research Institute New Jersey Medical School Rutgers University Newark, NJ United States

**Keywords:** COVID-19, COVID-19 triage, COVID-19 testing, SARS-CoV-2, feasibility study, National Dental Practice–Based Research Network, PBRN, dental practice, dental health, dentist, dentistry, safety, healthcare professional safety, health care, patient safety, dental healthcare staff

## Abstract

**Background:**

Dental practice has been greatly affected by the COVID-19 pandemic. As SARS-CoV-2 infection is transmitted by respiratory fluids, dental practice techniques, which include aerosol-generating procedures, can increase the risk of transmission causing heightened safety concerns for both dental health care workers (DHCWs) and patients. These concerns have resulted in the reduction in patient volume and the available workforce within dental practices across the United States. Standardized methods for COVID-19 triage and testing may lead to increased safety and perceptions of safety for DHCWs and their patients and promote willingness to provide and access oral health care services.

**Objective:**

This study is designed to develop procedures that test the feasibility of enhanced COVID-19 triage and testing in dental offices. It will provide preliminary data to support a larger network-wide study grant application aimed at developing protocols to address safety concerns of patients and DHCWs in a peri–COVID-19 pandemic era.

**Methods:**

The feasibility study is being conducted in 4 private dental practices, each of which has a dentist member of the National Dental Practice–Based Research Network. Participants include the DHCWs and patients of the dental practice. Study procedures include completion of COVID-19 triage, completion of COVID-19 testing (point-of-care [POC] or laboratory-based [LAB] SARS-CoV-2 viral, antigen, and antibody tests based on office designation), and administration of perception and attitude surveys for participating DCHWs and patients of the dental practice over a defined study period. The office designation and the participant’s role in the practice determines which testing protocol is executed within the office. There are 4 study groups following 4 distinct protocols: (1) POC DHCWs, (2) POC patients, (3) LAB DHCWs, and (4) LAB patients.

**Results:**

Data collection began in December of 2021 and concluded in March 2022. Study results are expected to be published in fall 2022.

**Conclusions:**

The results of this feasibility study will help identify the viability and functionality of COVID-19 triage and testing in dental practices and inform a larger network-wide study grant application that develops protocols that address safety concerns of patients and DHCWs in a COVID-19 environment.

**Trial Registration:**

ClinicalTrials.gov NTC05123742; https://clinicaltrials.gov/ct2/show/NCT05123742?term=NCT05123742

**International Registered Report Identifier (IRRID):**

DERR1-10.2196/38386

## Introduction

### Background

The COVID-19 pandemic has had a profound impact on the delivery of dental services in both the private and public sectors. The uniqueness of dental practice can increase the risk of transmission of SARS-CoV-2 and has heightened safety concerns for both dental health care workers (DHCWs) and patients, resulting in a reduction in available workforce and causing patients to delay essential and preventative care [1,2]. Moreover, as the COVID-19 pandemic evolved and transmission became more widespread, practice guidelines, and recommendations were ever changing, fueling a heightened level of uncertainty and caution [3]. The lack of standardized mitigation strategies, coupled with varying degrees of implementation within dental offices, further compromised the willingness of dental professionals to return to their dental practices and patients to seek care [4,5]. Establishing standardized COVID-19 triage and testing strategies in dental practices may ultimately increase safety and promote the feeling of safety for DHCWs and their patients.

### Uniqueness of Dental Practice

At the onset of the COVID-19 pandemic, the spread of this ubiquitous virus was first thought to be through droplets, but soon it became clear that the virus could be spread by aerosols, raising alarm for the dental community [6,7]. As many dental procedures generate significant aerosols through the use of high-speed handpieces and water coolant, the risk of aerosolized SARS-CoV-2 transmission significantly increases [8,9]. While it is certain that clinic dental personnel will be exposed to significant aerosol sprays derived from patients’ oral cavities, the extent to which dentally generated aerosols linger within the clinical space has not been clearly tested, and questions of duration of exposure and transmission risk remain [10]. As we learn more about COVID-19 and viral transmission, we understand that infection and spread of the virus is due to the viral load (or dose) and the time of contact [11,12]. Additionally, unlike many procedures in medicine, dental-patient contact requires close proximity between the patient and dental professionals [13]. A dental procedure can often take as long as 1 hour, and prolonged close dentist-patient contact is inevitable. As such, the likelihood of viral transmission is elevated in the dental setting and requires enhanced mitigation efforts in the treatment spaces to minimize disease transmission [6,14,15].

Beyond clinical treatment rooms, auxiliary space, including waiting rooms, bathrooms, and passageways are additional areas within offices that can be safety concerns [15]. The airflow in confined office spaces, where patients would wait for 30 minutes or more, can be hazardous if an asymptomatic but COVID-19–infected individuals are in close proximity to a susceptible patient [16]. Patients in a dental office can be at risk for infection that can have varied presentation and severity and may be life threatening especially in patients with pre-existing conditions [17]. Amid the pandemic, some offices chose to restrict patient access and eliminate waiting rooms altogether, while other offices staggered appointments and enhanced physical distancing measures. Despite the various methods employed, adequate ventilation and disinfection remain areas of concern [18].

### Solutions

One way of providing the security that dental offices are safe for both the dental professional and patient is by taking measures to reduce the possibility of anyone who harbors SARS-CoV-2 from entering the office, thereby limiting the risk of spread within the dental office. Mitigation strategies vary from office to office and often include identifying infected individuals through screening, most often through symptom questionnaires. As symptoms appear 2-14 days after infection (depending on a variety of factors including the variant), a triage questionnaire, based on symptomology, maybe only partially effective. Patient and DHCW triage are effective in identifying and isolating symptomatic individuals, though limitations exist by virtue of varying degrees and range of symptomatology [19]. The knowledge that asymptomatic and presymptomatic individuals may still transmit the virus suggests that solely triaging for symptoms may be inadequate to eliminate virus-infected patients from their offices [20]. More advanced diagnostic tools are necessary to identify infected individuals from initial onset of infection through their infectious period. Enhanced COVID-19 triage and testing could augment screenings and provide more reliable data to more accurately identify infected individuals and ultimately mitigate transmission amongst office personnel and patients.

Many aspects of testing have been highly debated in national forums, but little is known about the perceived value of COVID-19 testing in a dental practice and the willingness of DHCWs to implement such testing in their offices [21]. Furthermore, several challenges exist to routine and comprehensive testing in a dental office setting, including cost, ease of use, and turnaround time. Maximum practical utility would perhaps be derived from a simple, rapid, accurate, inexpensive point-of-care (POC) test that is not technically demanding, but such a test has not yet been validated.

### Importance of the Study

The effective use of testing and other modifications in dental practice could reduce the actual and perceived risk of COVID-19 transmission in a dental practice, facilitating dental health care providers’ and patients’ comfort with providing or seeking essential dental services. This protocol is designed to develop pragmatic procedures that address this serious problem and test the feasibility of COVID-19 triage and testing procedures in an active dental practice, utilizing the community of private practice researchers within National Dental Practice–Based Research Network (PBRN). The feasibility of implementing COVID-19–related testing and enhanced triage procedures in the dental setting will provide preliminary data to inform a larger network-wide study grant application that develops protocols that address safety concerns of patients and DHCWs in a COVID-19 environment.

The Pragmatic Return to Effective Dental Infection Control through Triage and Testing (PREDICT) PBRN Feasibility Study seeks to assess the following aims: (1) the willingness of DHCW and practice patients to participate in the research protocol; (2) the willingness and ability of practice patients and DHCWs to execute and follow through with triage, testing, and survey administration procedures; and (3) the ease of use of the Research Electronic Data Capture (REDCap) instruments, including interface access, survey design, and completion requirements for both the DHCW and practice patient end users. The PREDICT PBRN feasibility study will use qualitative and quantitative methods to collect outcomes measures and conduct data analysis. The ultimate goal of this research is to conduct a clinical study that assesses the impact of COVID-19 screening on dental practice including perceptions of safety and identifies the most efficient, acceptable, and effective workflow.

## Methods

### Ethics Approval

The study received institutional review board (IRB) approval through the Network single IRB (University of Alabama, IRB-300007026) and local context approval from Rutgers University IRB (Pro2021000968). The study was registered on ClinicalTrials.gov (NCT05123742).

### Study Design

This observational study is designed to assess the feasibility of implementing COVID-19–related testing and triage procedures in dental practices and focuses on increasing safety and perceptions of safety for the DHCWs and their patients. This study is being conducted in 4 dental practices of northeast region members of the PBRN (hereafter referred to as “the Network”) and participants include the DHCWs and patients of the practice [22]. Study procedures include completion of COVID-19 triage, completion of COVID-19–related testing (SARS-CoV-2 viral, antigen or antibody tests based on group designation), and administration of risk perception and attitude surveys for participating DCHWs and patients of the dental practice over a defined study period.

The office designation determines which testing protocol is executed within the office. Furthermore, a participant’s role in the practice further delineates the protocol. As such, there are 4 study groups following 4 distinct protocols reflecting POC and laboratory-based (LAB) testing procedures: (1) POC DHCWs, (2) POC patients, (3) LAB DHCWs, and (4) LAB patients (Table 1).

**Table 1:** Protocol by study group.

Study group			Protocol		COVID-19 tests (method)		Participants, n
**Point-of-care (POC)**							
	DHCW^a^ in POC office	POC DHCW		Antigen (nasal swab) + Antibody (capillary blood)		5-10 DHCWs	
	Patients in POC office	POC Patient		Antigen (nasal swab)		10 patients	
**Laboratory-based (LAB)**							
	DHCW in LAB office	LAB DHCW		Viral (saliva and tongue)+Antibody (capillary blood)		5-10 DHCWs	
	Patients in LAB office	LAB patient		Viral (saliva)		10 patients	

^a^DHWC: dental health care worker.

### Office Selection and Designation

This study is being conducted at 4 private practice dental offices, each of which employs at least one Network member dentist. Dentist members of the Network are first solicited for participation in this study. The dental office is enlisted if the Network dentist member and 4-9 additional office DHCWs of the practice are willing to participate and the Network member is willing to fulfil the responsibilities of the protocol, including enrolling and completing the assessment of 10 patients. The Network dentist member of the practice is responsible for indicating preference in office designation (POC or LAB) and the execution of the research study within the dental practice.

### Participants

#### Overview

The study population is drawn from Network dentist members, their coworkers, and patients seen in the dental office in which they work. The DHCW study population comprises dentists, hygienists, assistants, and office staff who work in an office with a Network member dentist. The patient study population is drawn from the dental practice of the Network member dentist.

#### Inclusion Criteria

A DHCW participant must meet the following criteria to be eligible to participate in the study: be aged 18 years or older, a Network member dentist, or work in a dental office with a Network member dentist who accepted study participation; understand the informed consent; provide a signed and dated informed consent form; have the capacity to understand all instructions for data collection instruments and be willing and able to comply with all study procedures, including COVID-19 testing; and be available for the duration of the study.

A patient participant must meet the following criteria to be eligible to participate in the study: be aged 18 years or older; be able to understand the informed consent; have access to a computer or electronic device with internet access; have the ability to complete consent and questionnaire on a computer or electronic tablet device; provide a signed and dated informed consent form; and have the capacity to understand all instructions for data collection instruments and be willing and able to comply with all study procedures, including having a COVID-19 test performed.

#### Exclusion Criteria

Participants are excluded if they participated in an earlier PREDICT feasibility study conducted at Rutgers University.

### Recruitment

Informed consent is obtained for each study participant.

#### DHCW Participants

Within 4 weeks prior to study initiation, DHCWs in the participating office are contacted to discuss the study and gauge participation interest. DHCWs who indicate interest are sent an email link for electronic consent on REDCap. The consent clearly outlines participant expectations and offers the opportunity for potential participants to contact the Network Node Coordinator for more information. DHCWs who elect to participate affirm their willingness to participate by electronically signing the consent form.

#### Patient Participants

Patients who have an upcoming appointment are solicited for participation. They receive an informational letter and follow-up phone call to discuss the study and determine their interest. Patients who indicate interest are sent an email link for electronic consent on REDCap. Patients who elect to participate affirm their willingness to participate by clicking the “I agree to take part in this study” button at the end of the consent form. Participation acceptance automatically triggers the Patient Pre-Visit Survey to be sent electronically to the enrolled patient.

### Study Schedule

**Figure 1:**
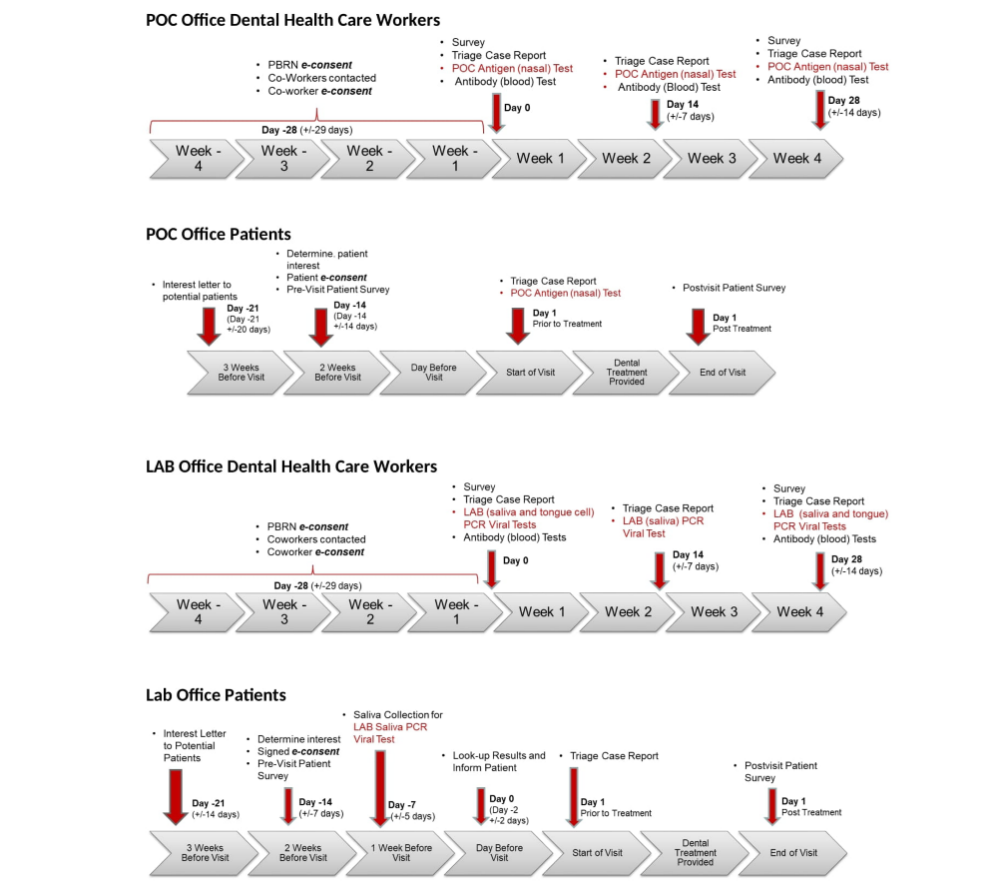
Study schedule for DHCW and patient participants in POC and LAB offices. DHCW: dental health care worker; LAB: laboratory; PBRN: National Dental Practice–Based Research Network; PCR: polymerase chain reaction; POC: point of care.

DHCWs actively engage in study procedures 3 times over a 1-month period, whereas patient participants engage in study procedures over a 2-week period prior to and at the end of their scheduled dental appointment. The 4 distinct protocols based on office designation and role in the dental practice are illustrated in Figure 1.

### Triage

In-office COVID-19 triage screening includes a series of questions related to symptoms commonly associated with SARS-CoV-2 infection (fever or chills, cough, shortness of breath or difficulty breathing, fatigue, muscle and body aches, headache, loss of taste, loss of smell, sore throat, congestion or runny nose, and nausea or vomiting), a temperature check, and an oxygen saturation measurement using a pulse oximeter. Results are documented on the triage case report. Patient participants undergo triage once the day of their appointment, whereas DHCW participants undergo triage in 3 intervals over a 1-month period.

### Testing

#### Overview

All testing materials are labeled and packaged in distinct participant kits, with explicit testing instructions and return shipping guidelines. Test kits for sample collection are shipped directly to the residence of patients participating in the LAB protocol. All other test kits, including those packaged for DHCWs in both the LAB and POC offices, as well as for patients in the POC offices, are shipped en masse to the dental office.

#### SARS-CoV-2 POC Tests

The Abbott BinaxNOWTM Covid-19 antigen card used in this study is specific for SARS-CoV-2 nucleocapsid protein antigen and used to determine SAR-CoV-2 infection. As part of both the DHCW and Patient POC office study protocols, participants will undergo a SARS-CoV-2 POC test in the dental office. A nasal swab is swept inside the participant’s nose (not beyond the nares) to collect the specimen for the POC test. The office staff processes the specimen and reads the results within 15 minutes.

#### PCR Viral Tests

The polymerase chain reaction (PCR) test is a molecular test that analyzes specimens from a subject’s nose or mouth to detect the RNA of SARS-CoV-2—the virus that causes COVID-19. This test is used as an indicator of COVID-19 infection. Patient participants following the LAB protocol are asked to provide a saliva sample using the saliva collection kit and mail it to the laboratory for processing 1 week prior to the scheduled dental visit. DHCW participants are required to provide a similar saliva sample 3 times during their month-long participation. In addition, DHCWs are asked to provide tongue specimens, collected using a cytology brush and collection medium, twice during their participation. Both saliva and tongue samples are mailed to Rutgers University laboratories for processing.

Specifically, RNA extraction will be performed and the total RNA concentrations for saliva, tongue, and nasal samples will be determined using a Nanodrop One machine (Thermo Scientific).

#### Enzyme-Linked Immunosorbent Assay Antibody Tests

This antibody test utilizes peripheral blood to detect immunoglobulin G (IgG) and immunoglobulin M (IgM) antibodies against the SARS-CoV-2 antigen, which indicates a history of SARS-CoV-2 infection. All DHCWs in either the POC or LAB protocol undergo an enzyme-linked immunosorbent assay (ELISA) antibody test at 2 intervals during the study. Following finger puncture with a lancet device, peripheral blood is collected utilizing a volumetric absorptive microsampler (Mitra Collection Kit, Neoteryx). The microsamplers are packaged by the DHCW following the manufacturer’s instructions and placed in the practice collection box for shipment to the testing laboratory. Prior to ELISA, fluid is eluted from the microsamplers. Detection of IgM and IgG antibodies directed against the receptor binding domain of the SARS-CoV-2 Spike protein is performed in accordance with published ELISA protocols [23].

### Data Management

Study data and participant consent were collected and managed using the REDCap software [24]. REDCap is a secure, web-based software platform designed to support data capture for research studies. All data elements are being entered directly into the REDCap system, including subject survey responses, triage case reports, and testing orders and results.

### Study Surveys

DHCW participants complete 3 surveys over a 1-month period: DHCW Start-of-Study Survey, DHCW End-of-Study Survey, and the Participation Survey. The DHCW Start-of-Study and DHCW End-of-Study surveys include the validated 6-question Safety Culture Evaluation Survey aimed at assessing workplace safety. Specifically, the DHCW Start-of-Study Survey administered on day 1 includes questions related to demographics, personal protective equipment (PPE) used in the office, work practice controls used in the office, importance of triage and testing, importance of PPE measures, perceptions of safety and comfort in the workplace, safety culture in the office, SARS-CoV-2 testing preferences, dentists’ role in SARS-CoV-2 testing, and willingness to test in the dental office. The DHCW End-of -Study Survey administered on day 28 includes questions related to the importance of triage and testing, importance of PPE measures, perceptions of safety and comfort in the workplace, safety culture in the office, SARS-CoV-2 testing preferences, dentists’ role in SARS-CoV-2 testing, willingness to test in the office, and vaccinations.

Patient participants are asked to complete 3 surveys: the Patient Pre-Visit Survey, Patient End-of-Visit Survey, and Participation Survey. Launched automatically after the completed consent approximately 2 weeks prior to their dental visit, patient participants are asked to complete the Pre-Visit Survey, which explores COVID-19 exposure and vaccination history, perceptions of safety and comfort, reasons for delaying dental care, concerns about returning to dental care, safety precautions valued, importance of triage and testing, and demographics. Following their dental treatment, patient participants will complete 2 electronic surveys. The Patient End-of-Visit Survey explores perceptions with testing preferences, PPE observed, environmental controls observed, concerns about returning to dental care, safety precautions valued, importance of triage and testing, likelihood of reporting symptoms, dentists’ role in COVID-19 testing, and vaccinations.

All participants enrolled in the feasibility study are asked to complete a participation survey, which will solicit feedback related to study participation including survey and testing logistics, preferences, and testing ease.

### Statistical Analysis

#### Sample Size Considerations

This is a feasibility study to assess viability of conducting study procedures within dental practices and refine study logistics for a larger multisite clinical trial. No sample size calculations were performed, but the results will be used for sample size calculations for future studies. As the Network attracts both solo practitioners and practitioners in multi-dentist offices, pilot sizes were set to 5-10 DHCWs. This range allows for participation of smaller offices as well as those with greater numbers of dentists and auxiliary staff. Ten patient participants were enrolled to adequately test office workflows in terms of both time and space availability within a functioning practice.

#### Analyses for Aim 1: Willingness to Participate

Willingness to participate is an important determinant in deciding whether dental offices, DHCWs, and patients would be willing to participate in a large-scale study. Analysis of the following outcomes measures will be conducted: (1) proportion of DHCWs who agree to participate among those who were approached for participation, (2) proportion of patients who agree to participate among those patients who were approached for participation, and (3) percentage of individuals who indicate the opportunity to ask questions during the consent process. These first 2 measures will be assessed at the time of consent, while measure number 3 data is derived from the Patient Participation Survey.

#### Analyses for Aim 2: Willingness or Ability to Follow Through With Triage, Testing, and Survey Administration Procedures

Determination of the willingness and ability to follow through with triage, testing and survey administration procedures are important for refining the survey procedures. Analysis of the following outcome measures for both patient and DHCW participants will be conducted: (1) percentage of individuals who complete the study, (2) percentage of individuals who complete the surveys, (3) percentage of activities occurring within each defined window, and (4) percentage of individuals who feel complying with testing (saliva, PCR, POC, and ELISA antibody tests) procedures was easy. Compliance will include specimen collection, specimen preparation for shipping, specimen storage, timeliness of results, and reporting of results.

#### Analyses for Aim 3: Ease of Use With the REDCap Survey Instruments

Determination of the ease of use and completeness of the REDCap instruments should enable refinement of the system. We will conduct analysis for the following outcomes measures for both patient and DHCW participants: (1) percentage of individuals who feel surveys are easy to complete owing to the administration method, (2) percentage of individuals who feel that the survey questions are understandable, and (3) percentage of individuals who complete each of the following surveys: DHCW Start-of-Study Survey, DHCW End-of-Study Survey, Pre-Visit Patient Survey, End-of-Visit Patient Survey, and the Triage Case Report.

## Results

The PREDICT study was funded in September 2020. Data collection began in December 2021 and concluded in March 2022. In total, 30 DHCW and 45 patient participants consented. Following data analysis, study results are expected to be published in fall 2022. The results from this study will also provide feasibility data to support a larger network-wide study grant application aimed at developing protocols to address safety concerns of patients and DHCWs in a peri–COVID-19 pandemic era. This project will also inform and shape responses to future pandemics.

## Discussion

### Expected Findings

The PREDICT study sought to develop and assess procedures for improved COVID-19 triage and testing in dental practices to increase safety and perceptions of safety of DHCWs and their patients. As testing is an effective mitigation strategy that has become commonplace in the COVID-19 era, it is anticipated that both DHCWs and patients would not only agree to participate, but also effectively carry out testing and triage procedures. With cell phones and other electronic devices used as widely accepted communication tools, it is expected that participants will engage in electronic surveys through the REDCap interface with ease.

### Study Strengths and Limitations

This novel study design engages both the office personnel and patients through targeted testing and triage to synergistically enhance feelings of safety and increase willingness to return as employees and as patients. The study team recognized that in order to alter the overall perception of safety in a meaningful way, one could not focus on a single group but rather on the office collective. Typical dental practices employ a small number of office staff, who perform a myriad of functions, from delivery of care to front desk and patient billing. All roles are essential for the success of the practice. Absences related to COVID-19 could significantly hamper operations and limit patient flow, ultimately leading to decreased production, revenue, and care delivery.

As time away from chair-side dentistry can directly affect revenue, researchers considered the cost-benefit ratio of triage and testing within an office. In designing the protocol, the study team was cognizant of dental office dynamics and workflow. With rapid patient turn-over and limited physical space, triage and testing needed to be efficient and cost-effective. Efforts to maximize protocol efficiency are the focus of the unique study design. Several initial study procedures for patient participants are conducted electronically outside the dental office prior to the patient dental visit, minimizing extended in-office time. Through the REDCap interface, patients’ consent is obtained at home with electronic signatures captured via REDCap. Participation acceptance triggers the automatic launch of the electronic Patient Pre-visit Survey, allowing them to complete the survey unimpeded, within the comfort of their own home. Similarly, saliva specimen collection for LAB patient testing occurs at home, significantly reducing the burden on office staff. It became clear that a feasibility study was necessary help identify viability and functionality of testing, as well as the preferred method of testing in a dynamic dental practice. In a larger-scale trial, there is a more comprehensive cost-benefit analysis planned to look at specific time requirements for testing related to diversion of staff support, productivity, and cost allocation to patients.

Limitations to protocol implementation were identified and primarily revolved around Network member dentist training. The complexity of the study warranted several training sessions, and practitioner availability for real-time training was limited. While training for this feasibility study was conducted contemporaneously via a digital platform, it was apparent that this method would be inefficient in a larger-scale trial. A concerted effort to ensure training efficiency would be required for a larger, nationwide clinical trial implemented in dental practices across multiple states and time zones. The use of prerecorded training modules would help alleviate the time burden placed on practitioners, enhance the understanding of the protocol, and eliminate questions through on-demand viewing capabilities.

Utilizing a multilocation practice as a designated office (POC or LAB) added a layer of complexity to the execution of this study protocol related to subject specimen kits. During this feasibility study, study kits were sent to one office location. In our larger multisite practice, these kits required to be transferred from one office to another within a practice. Similarly, after specimen collection, completed study kits needed to be collated prior to shipping them back to the laboratory for processing. In a larger-scale trial, study kits for a multi-office practice should be sent separately, reducing the time and effort burden on office staff.

### Conclusions

The PREDICT PBRN feasibility study will assess the viability and workability of COVID-19 triage and testing within dental practices. Our results will influence protocols proposed for a larger network-wide study grant application that addresses safety concerns of patients and DHCWs in a COVID-19 environment. Establishing enhanced triage and testing protocols within dental offices not only has the potential to not only increase willingness of patients and staff to return to work during the COVID-19 pandemic but also may have utility in the event of another disease outbreak.

## Abbreviations

DHCW: dental health care worker
ELISA: enzyme-linked immunosorbent assay
IgG: immunoglobulin G
IgM: immunoglobulin M
IRB: institutional review board
LAB: laboratory-based
PBRN: National Dental Practice–Based Research Network
PCR: polymerase chain reaction
POC: point of care
PPE: personal protective equipment
PREDICT: Pragmatic Return to Effective Dental Infection Control through Triage and Testing
REDCap: Research Electronic Data Capture

## Data Availability

The data sets generated or analyzed in this study are available from the corresponding author on reasonable request.
